# Partial deletion in the cuticular protein gene *BmorCPR2* results in a body shape mutant in silkworm, *Bombyx mori* L. (Lepidoptera: Bombycidae)

**DOI:** 10.1093/jisesa/ieag089

**Published:** 2026-08-21

**Authors:** Juan Sun, Min Liu, Xin Zheng, Gui Ouyang, Anli Chen, Heying Qian

**Affiliations:** Key Sericultural Laboratory of Shaanxi, Ankang University, Ankang, Shaanxi, China; College of Biotechnology, Jiangsu University of Science and Technology, Zhenjiang, Jiangsu, China; The Sericultural and Apicultural Research Institute, Yunnan Academy of Agricultural Sciences, Mengzi, Yunnan, China; Key Sericultural Laboratory of Shaanxi, Ankang University, Ankang, Shaanxi, China; College of Biotechnology, Jiangsu University of Science and Technology, Zhenjiang, Jiangsu, China; Key Sericultural Laboratory of Shaanxi, Ankang University, Ankang, Shaanxi, China; College of Biotechnology, Jiangsu University of Science and Technology, Zhenjiang, Jiangsu, China; Key Sericultural Laboratory of Shaanxi, Ankang University, Ankang, Shaanxi, China; College of Biotechnology, Jiangsu University of Science and Technology, Zhenjiang, Jiangsu, China

**Keywords:** lepidopteran model insect, cuticular mutation, functional verification

## Abstract

The *Bombyx mori* L. (Lepidoptera: Bombycidae) is a significant economic insect used for silk production. A novel body shape mutant, *stony^^sunken^* (*st^^sk^*), that exhibits a sunken intersegmental membrane was isolated from the wild type of *st^^sk^* (WT-n08). Investigation indicated that the mutation had no significant effect on its growth and development. To elucidate the molecular mechanism underlying this body shape mutant, genetic analysis, positional cloning, and the CRISPR/Cas9 gene editing system were performed. Genetic analysis demonstrated that the mutant trait in *st^^sk^* is controlled by an autosomal recessive gene and follows Mendelian inheritance. Positional cloning showed that a putative cuticular protein gene, *BmorCPR2* on chromosome 8, was the candidate gene. Sequencing analysis revealed partial deletion of *BmorCPR2* exon 2 and intron 2 sequences occurred and subsequently resulted in the premature termination of gene expression. Knock-out of *BmorCPR2* using the CRISPR/Cas9 gene editing system led to the sunken intersegmental membrane phenotype. These findings highlight the essential role of *BmorCPR2* in silkworm cuticular formation, providing a foundation for further research on cuticular protein function.

## Graphical abstractGraphical abstract

**Graphical Abstract:**
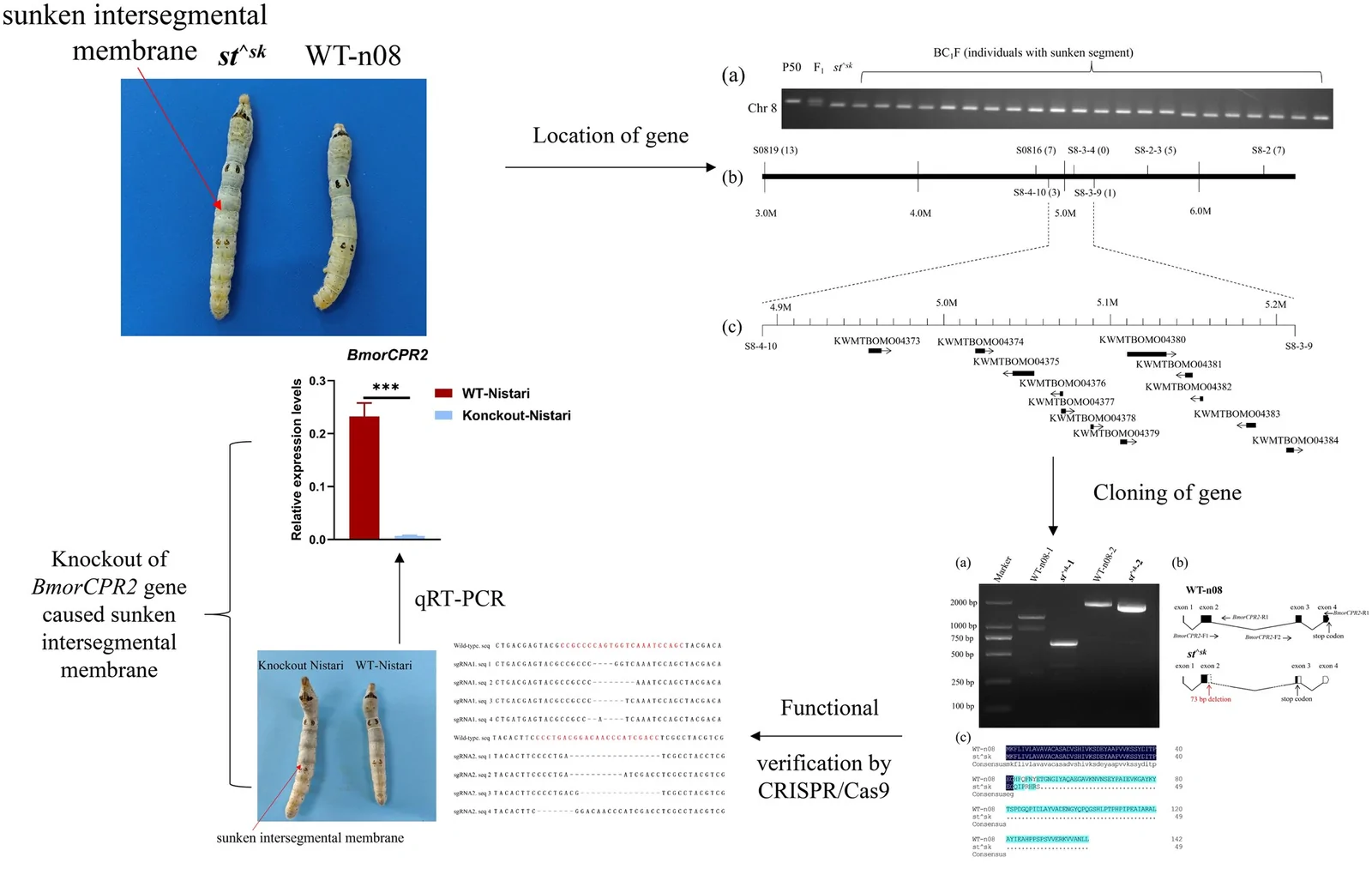

## Introduction

*Bombyx mori* L. (Lepidoptera: Bombycidae) is an important economic insect mass-reared in over 70 countries for silk production with rich genetic mutation systems. The body shape mutant *Moniliform 2* (*mf-2*) was discovered in 2009 (Dai et al. 2009), and its larvae exhibit deep constrictions in the intersegmental membranes with tightly contracted body segments, giving it a bead-like appearance. Its body is slender and rigid, prone to rectal prolapse, and demonstrates relatively slow growth. After that, other body type mutants have also been reported, such as *contractile* (*cot*), *tubby* (*tub*), *Bamboo* (*Bo*), *stick* (*sk*), etc (Nie et al. 2015, Wang et al. 2018, Xiong et al. 2018, Tan et al. 2022). Wang et al. used positional cloning and RNA interference to determine that the specific deletion of 312 bp of *Bmscarface* changed the spatial structure of the protein, resulting in the tub mutant larvae’s body type being short and fat with a spindle shape (Wang et al. 2018). The *Bo* mutant larvae exhibit a bamboo-shaped body plan characterized by thoracic expansion and a slender yet sclerotized abdomen, accompanied by reduced cuticular pigmentation. Through the application of positional cloning, RNA interference, and CRISPR/Cas9 gene editing techniques, it has been conclusively demonstrated that the *Bo* phenotype results from mutations in the cuticular protein gene BmorCPH24 (Xiong et al. 2018).

Insect cuticles play a crucial role in environmental adaptation by providing protection, structural support, and attachment points for internal muscles and organs, thereby influencing movement and flight (Delon and Payre 2004, Moussian et al. 2005). Chitin is polymerized from N-acetyl-D-glucosamine by β-(1,4) glycosidic bonds, forming a highly stable and insoluble protective layer. Cuticular proteins (CPs) are one of the important components of the cuticle, exhibit diverse structures and properties, and constitute over 1% of the total protein-coding genes in the insect genome (Futahashi et al. 2008), highlighting their significant role in physiological processes such as growth, reproduction, and environmental adaptation (Qiu et al. 2025). A total of 236 CPs have been identified in *B. mori*. These CP genes are classified into 12 families (CPR, CPT, CPAP1, CPAP3, CPF, CPFL, CPCFC, CPLCP, CPLCA, CPG, 18 aa, CPH) (Fu et al. 2023). The cuticular proteins of the CPR family, which is the largest family, are the most widely distributed and the largest in number (Jasrapuria et al. 2010). They play an important role in the entire life cycle of insects (Yang et al. 2017). Knockdown of *CfCPRR2-1* significantly decreased phosphine resistance in *Cryptolestes ferrugineus* (Stephens, Coleoptera: Laemophloeidae) (Chen et al. 2023), while suppression of *TcCPR62* disrupted elytral development in *Tribolium castaneum* (Herbst, Coleoptera: Tenebrionidae) (Wang 2022). In *Bombyx mori*, the coding sequence mutant of *BmorCPR2* led to the loss of the coding product’s ability to bind to chitin, resulting in the tight bodies of the *stony* (*st*) mutant larvae, protruding internode folds, and serious defects in larval adaptability (Qiao et al. 2014). These findings demonstrate the indispensable role of cuticular proteins in insect development and survival.

**Fig. 1.**
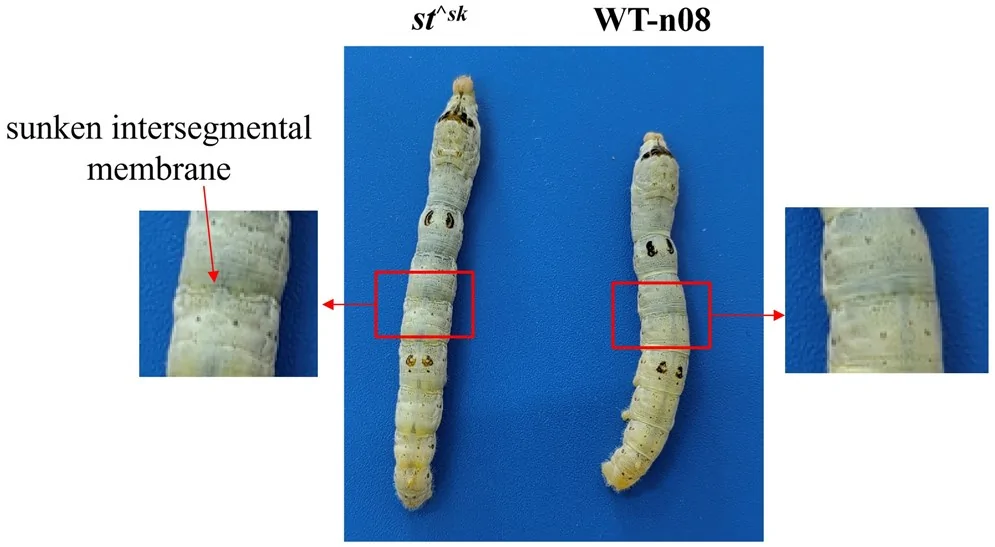
Phenotypes of *st^^sk^* and WT-n08. Intersegmental membrane of the *st^^sk^* mutant was sunken, as compared to the wild-type WT-n08.

In this study, a novel body shape mutant in *B. mori*, *stony^^sunken^* (*st^^sk^*), was reported, whose larvae exhibit depressed phenotypes in cuticular intersegmental membranes (Fig. 1). However, their behavioral patterns and developmental progression displayed no significant alterations. The aim of this study was to elucidate the molecular mechanism underlying this body shape mutant. Through positional cloning and CRISPR/Cas9 gene editing, a putative cuticular protein gene, *BmorCPR2*, was identified as the gene responsible for this phenotype. This study enhances the understanding of the genetic and molecular regulation of cuticular protein expression and underscores the critical role of epidermal proteins in maintaining the structural integrity of the insect exoskeleton.

## Materials and Methods

### Silkworm Strains

Wild-type p50 was maintained at the Sericultural and Apicultural Research Institute, Yunnan Academy of Agricultural Sciences. WT-n08 and its cuticular mutant strain *st^^sk^*, along with the polyvoltine diapause-free strain Nistari and its transgenic variant carrying the Cas9 gene (Nistari-Cas9), were preserved at the Sericultural Research Institute of Jiangsu University of Science and Technology. All *B. mori* larvae were reared on fresh mulberry leaves at 25 ± 0.5 °C under 75% to 80% humidity.

### Genetic Analysis of *st^^sk^*

F_1_ generation was obtained by crossing p50 and *st^^sk^*, followed by self-crossing to produce the F_2_ generation. Additionally, backcrosses (BC_1_F and BC_1_M) were performed. Phenotypic segregation ratios were calculated to determine inheritance patterns.

### Gene Mapping

Genomic DNA of the midgut of p50, *st^^sk^*, F_1_, BC_1_F, and BC_1_M was extracted using a cell/tissue genomic DNA extraction kit (BioTeke, China). SSR primers were designed according to the constructed silkworm SSR marker linkage map (Miao et al. 2008), and additional SSR markers were designed using SSR Hunter 1.3. PCR reaction system (20 μL): 10× buffer, 2 μL; dNTP, 0.5 μL; rTaq, 0.2 μL; Forward and reverse primers (10 μmol/L), 1 μL each; genomic DNA, 1 μL; and ddH_2_O, 14.3 μL. The PCR reaction program was as follows: 94 °C for 5 min; 32 cycles at 94 °C for 30 s, 57 °C for 25 s, 72 °C for 25 s; 72 °C for 5 min. PCR products were separated by 2% agarose gel electrophoresis to identify polymorphisms. Given that the homologous chromosomes of female silkworms do not exchange, linkage group determination utilized 22 BC_1_F mutant individuals, while 200 BC_1_M mutants facilitated fine mapping.

### Gene Cloning

Specific primers (Supplementary Table S1) were designed based on target gene sequences obtained from Kaikobase (https://kaikobase.dna.affrc.go.jp). The total RNA of WT-n08 and *st^^sk^* was extracted using RNAiso Plus (TaKaRa, Japan). Extracted RNA was reverse-transcribed using a PrimerScript RT reagent kit with gDNA eraser (TaKaRa, Japan). Reaction system was divided into two steps (20 μL): the first step (10 μL): 5 × gDNA Eraser Buffer, 2 μL; gDNA Eraser, 1 μL; Total RNA, 1 μg; RNase Free dH_2_O, up to 10 μL; 42 °C for 2 min; 4 °C for ∞; the second step (20 μL): the reaction solution of the first step, 10 μL; PrimeScript Enzyme Mix I, 1 μL; RT Primer Mix, 1 μL; 5 × PrimeScript Buffer 2, 4 μL; RNase Free dH_2_O, 1 μL; 37 °C for 15 min; 85 °C for 5 s; The obtained cDNA was used as templates for PCR amplification. Amplified products were analyzed by 1% agarose gel electrophoresis, cloned into a pMD19-T vector, transformed into *Escherichia coli* competent cells, and sent to Sangon Biotech Co., Ltd. for sequencing. Sequence analysis was performed using BioEdit.

### Construction of Target Gene sgRNA Vector and Microinjection

The target gene sequence was amplified using Nistari genomic DNA as a template and sequenced to compare whether there were any differences between the sequence and the reference sequence. CRISPRdirect (http://crispr.dbcls.jp) was utilized to search for two potential sgRNA targets within the ORF region of the sequenced target gene, and sgRNA primers (Supplementary Table S1) were designed based on the target gene sequence and the transgenic vector pXL (IE1-DsRed). The sgRNA sequence was inserted into the pXL (IE1-DsRed) vector via homologous recombination to obtain the target plasmid. The sgRNA transgenic plasmid was driven by the U6 promoter, and the IE1 promoter was used for the red fluorescence reporter gene expression. The Cas9 promoter was also IE1 (Supplementary Fig. S1). The CRISPR/Cas9 plasmid map is shown in Supplementary Fig. S1. Each 50 μL PCR reaction system included 25 μL Primer STAR Premix (2×), 1.5 μL forward primer (10 μmol/L), 1.5 μL reverse primer (10 μmol/L), 2 μL pXL (IE1-DsRed), and 20 μL RNase-free H_2_O. The reaction conditions were as follows: 94 °C for 2 min, 31 cycles at 94 °C for 20 s, 56 °C for 20 s, 72 °C for 40 s, and an extension at 72 °C for 7 min. The PCR product was purified, and homologous recombination was performed to insert the PCR product of the two sgRNA sites into the pXL (IE1-DsRed) vector, which had been linearized by double digestion with SalI and NheI, following the In-Fusion Snap Assembly Master Mix kit (TaKaRa, Japan) instructions.

The recombinant vector was transformed into competent *E*. *coli* cells, and after identification by bacterial liquid PCR, the samples were sent for sequencing. The recombinant plasmid was extracted using the QIAGEN Plasmid Midi Kit (QIAGEN, Germany). Both the recombinant and helper plasmids were microinjected into 4- to 6-hour-old non-diapausing Nistari silkworm eggs.

### Knockout Validation

The G_0_ generation was reared under normal temperature and humidity conditions. G_0_ individuals were self-crossed to produce the G_1_ generation. Newly hatched larvae from the G_1_ generation were examined under a fluorescent stereomicroscope, and individuals with red fluorescence were selected. The moths of the positive individuals were crossed with the Cas9 transgenic silkworm to produce the G_2_ generation. Newly hatched silkworms from the G_2_ generation were again observed under a fluorescent stereomicroscope, and individuals emitting both red and green fluorescence were selected.

Fifth-instar larvae exhibiting sunken intersegmental membranes were chosen for genome DNA extraction using a cell/tissue genome DNA extraction kit (BioTeke, China). The sgRNA target sequences were analyzed using knockout detection primers (Supplementary Table S1). Each 20 μL PCR reaction system contained 2 μL of 10× LA Taq buffer, 3 μL of dNTP mixture, 0.2 μL of LA Taq enzyme, 1 μL of forward primer, 1 μL of reverse primer, 1 μL of DNA template, and 11.8 μL of ddH_2_O. The PCR reaction procedure was as follows: 94 °C for 5 min, 31 cycles at 94 °C for 30 s, 55 °C for 30 s, 72 °C for 30 s, and extension at 72 °C for 10 min. The PCR products were cloned into the pMD19-T vector, and single clones were selected for sequencing.

### Quantitative Reverse Transcription PCR

Total RNA was extracted from the cuticle of both wild-type Nistari and G2 individuals with sunken intersegmental membranes using RNAiso Plus (TaKaRa, Japan). RNA was reverse-transcribed using a PrimerScript RT reagent kit with gDNA eraser (TaKaRa, Japan) according to the manufacturer’s protocol. The cDNA was diluted fivefold with ddH_2_O. Each 20 μL qRT-PCR reaction contained 10 μL of iTaq Universal SYBR Green Supermix (Bio-Rad, USA), 0.5 μL of each specific primer, 1 μL of cDNA template, and 8 μL of ddH_2_O, and three replicates for each reaction. qRT-PCR was carried out on a CFX96 PCR system (Bio-Rad, USA) using a two-step reaction protocol: 40 cycles at 94 °C for 5 s and 60 °C for 1 min. The *B. mori* actin 3 gene (GenBank ID: NM_001126254) was used as a reference gene to eliminate bias in samples, and qRT-PCR data were analyzed using the 2^-ΔΔ^^*Ct*^ method (Livak and Schmittgen 2001). The primers used for qRT-PCR were listed in Supplementary Table S1.

## Results

### Genetic Analysis and Economic Traits of *st^^sk^*

Genetic analysis revealed that the body shape of F_1_ individuals was normal. Self-crossing of F_1_ individuals led to offspring that segregated for normal and sunken individuals in a 3:1 ratio. Backcross populations exhibited a 1:1 segregation ratio between normal and sunken individuals (Table 1), confirming that the *st^^sk^* phenotype was controlled by a recessive gene located on an autosome.

**Table 1.** Trait segregation of each generation between *st^^sk^* and p50

Hybrid form	Generation	Number of normal individuals	Number of mutants	Total number of silkworms	Separation ratio	*χ* ^2^	*P-*value
***st^^sk^*♀×p50♂**	F_1_	318	0	318	N	–	
**p50♀×*st^^sk^*♂**	F_1_	394	0	394	N	–	
**(*st^^sk^*♀·p50♂)♀× (*st^^sk^*♀·p50♂)♂**	F_2_	279	103	382	3:1	0.78	0.376
**(p50♀·*st^^sk^*♂)♀× (p50♀·*st^^sk^*♂)♂**	F_2_	311	116	427	3:1	1.07	0.301
**(*st^^sk^*♀·p50♂)♀×*st^^sk^*♂**	BC_1_F	206	185	391	1:1	1.13	0.288
**(p50♀·*st^^sk^*♂)♀×*st^^sk^*♂**	BC_1_F	205	219	424	1:1	0.46	0.497
***st^^sk^*♀× (*st^^sk^*♀·p50♂)♂**	BC_1_M	171	156	327	1:1	0.69	0.407
***st^^sk^*♀× (p50♀·*st^^sk^*♂)♂**	BC_1_M	173	159	332	1:1	0.59	0.442

The values in the table are the average values of three batches, *P* > 0.05 by *χ*^2^ tests, “N” means no separation of traits.

A comparative analysis of nine important economic traits between WT-n08 and *st^^sk^* showed no statistically significant differences (Table 2), indicating that the gene mutation did not affect the economic traits. These findings established that the *st^^sk^* mutant had no obvious adaptability defects.

**Table 2.** Comparison of the economic traits between WT-n08 and *st^^sk^*

Economic traits	WT-n08	*st^^sk^*	*P*
**Average number of eggs laid**	417 ± 12.85	416 ± 8.73	0.944
**Larval rate/%**	97.23 ± 0.02	97.33 ± 0.11	0.199
**Dead silkworm cocoons rate/%**	0.55 ± 0.02	0.56 ± 0.03	0.252
**Duration of 5th instar/(d:h:m)**	5:12:21	5:12:25	–
**Duration of all-instars/(d:h:m)**	20:12:44	20:12:42	–
**Larva-pupa rate/%**	96.71 ± 0.02	96.63 ± 0.09	0.216
**Double cocoons rate/%**	0.27 ± 0.01	0.26 ± 0.01	0.288
**Cocoon weight/g**	1.28 ± 0.01	1.26 ± 0.02	0.219
**Cocoon shell weight/g**	0.23 ± 0.01	0.22 ± 0.01	0.288
**Cocoon shell rate/%**	15.6 ± 0.26	15.4 ± 0.26	0.407

The data in the table represent the mean ± standard deviation of three replicates.

### Positional Cloning and Candidate Gene Sequence Analysis

**Fig. 2.**
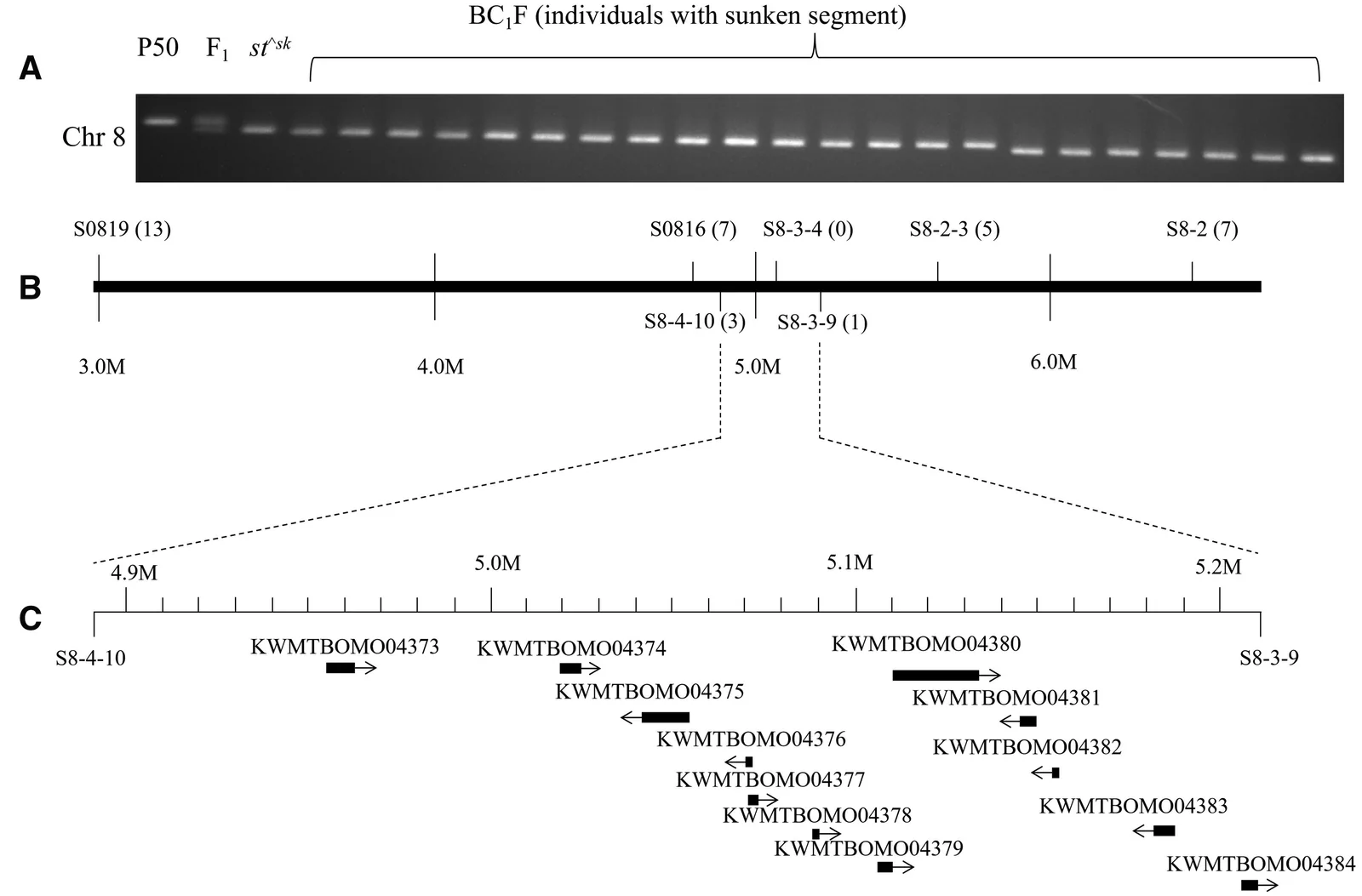
Mapping linkage map of mutant genes. A) Linkage analysis of the 8th linkage group of *st^^sk^*. Lane 1: p50, lane 2: F_1_, lane 3: *st^^sk^*, and lanes 4 to 25: BC_1_F individuals with sunken intersegmental membranes. B, C) Fine mapping narrowed the region tightly linked to the *st^^sk^* phenotype between SSR markers S8-4-10 and S8-3-9. The numbers in parentheses indicate the number of recombined individuals in the BC_1_M population.

Given the lack of meiotic recombination in female silkworm chromosomes, 22 individuals from the BC_1_F population were genotyped with polymorphic SSR molecular markers from each autosome. The PCR bands of the 22 BC_1_F individuals with sunken intersegmental membranes, amplified with SSR markers on chromosome 8, matched those of *st^^sk^* (Fig. 2A), while the PCR bands of other autosomal markers were distributed irregularly. Thus, the gene responsible for the sunken phenotype of *st^^sk^* was mapped to the 8th linkage group of *B. mori*.

**Fig. 3.**
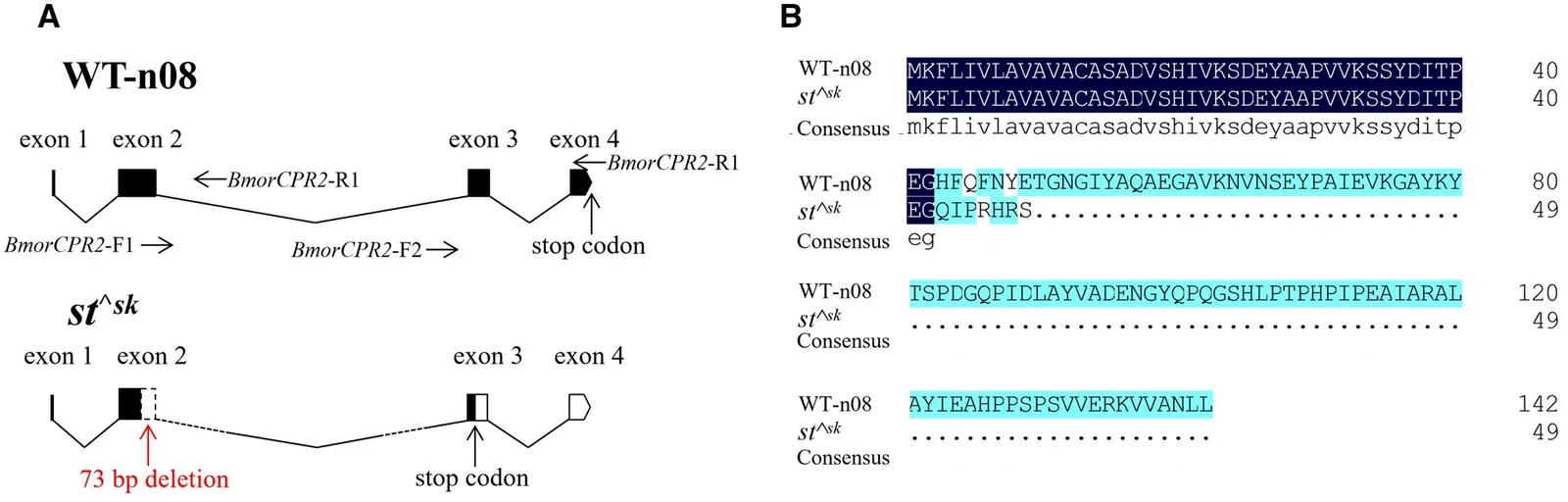
Sequence alignment of *BmorCPR2*. A) Exon structures of *BmorCPR2* in wild-type (WT-n08) and *st^^sk^* mutants. In *st^^sk^* mutants, a partial deletion in *BmorCPR2* exon 2 and intron 2 was observed, resulting in a stop codon in the functional region and premature termination of gene expression. Exons are indicated by black boxes, and deletion sequences are marked with dashed lines. Arrows indicate the position of primers listed in Table S1. B) Amino acid sequence alignment of the *BmorCPR2* gene between WT-n08 and *st^^sk^*.

To narrow down the location, seven SSR molecular markers on the 8th linkage group were further analyzed (Table 3). The results suggested that the mutant gene was located between markers S8-4-10(3) and S8-3-9(1) (Fig. 2B and C), spanning approximately 300 kb with 12 candidate genes: KWMTBOMO04383 through KWMTBOMO04384. Analysis of the functions of these candidate genes using the silkworm database (https://kaikobase.dna.affrc.go.jp/) revealed that KWMTBOMO04383 (a hypothetical protein gene) and KWMTBOMO04384 (a putative cuticular protein gene, *BmorCPR2*) are associated with insect cuticular proteins. Sequence analyses of the CDS region showed no change in KWMTBOMO04383, but a 73 bp deletion in exon 2 and partial deletion in intron 2 of KWMTBOMO04384 (*BmorCPR2*) were found that resulted in a frameshift mutation and premature termination of gene expression (Fig. 3 and Supplementary Fig. S2). This loss of function in *BmorCPR2* may be responsible for the sunken intersegmental membrane in *st^^sk^*.

**Table 3.** Fine mapping primers of SSR markers of the linkage groups of *Bombyx mori*

Mark symbols	Location of the marker on chromosome 8	Exchange number/total number of BC_1_M
**S0819**	3005758–3006075	13/200
**S0816**	4808286–4808451	7/200
**S8-4-10**	4884773–4884907	3/200
**S8-3-4**	5047723–5047932	0/200
**S8-3-9**	5213836–5214024	1/200
**S8-2-3**	5623617–5623720	5/200
**S8-2**	6660848–6661116	7/200

### Acquisition of Transgenic Silkworm and Knockout Validation

**Fig. 4.**
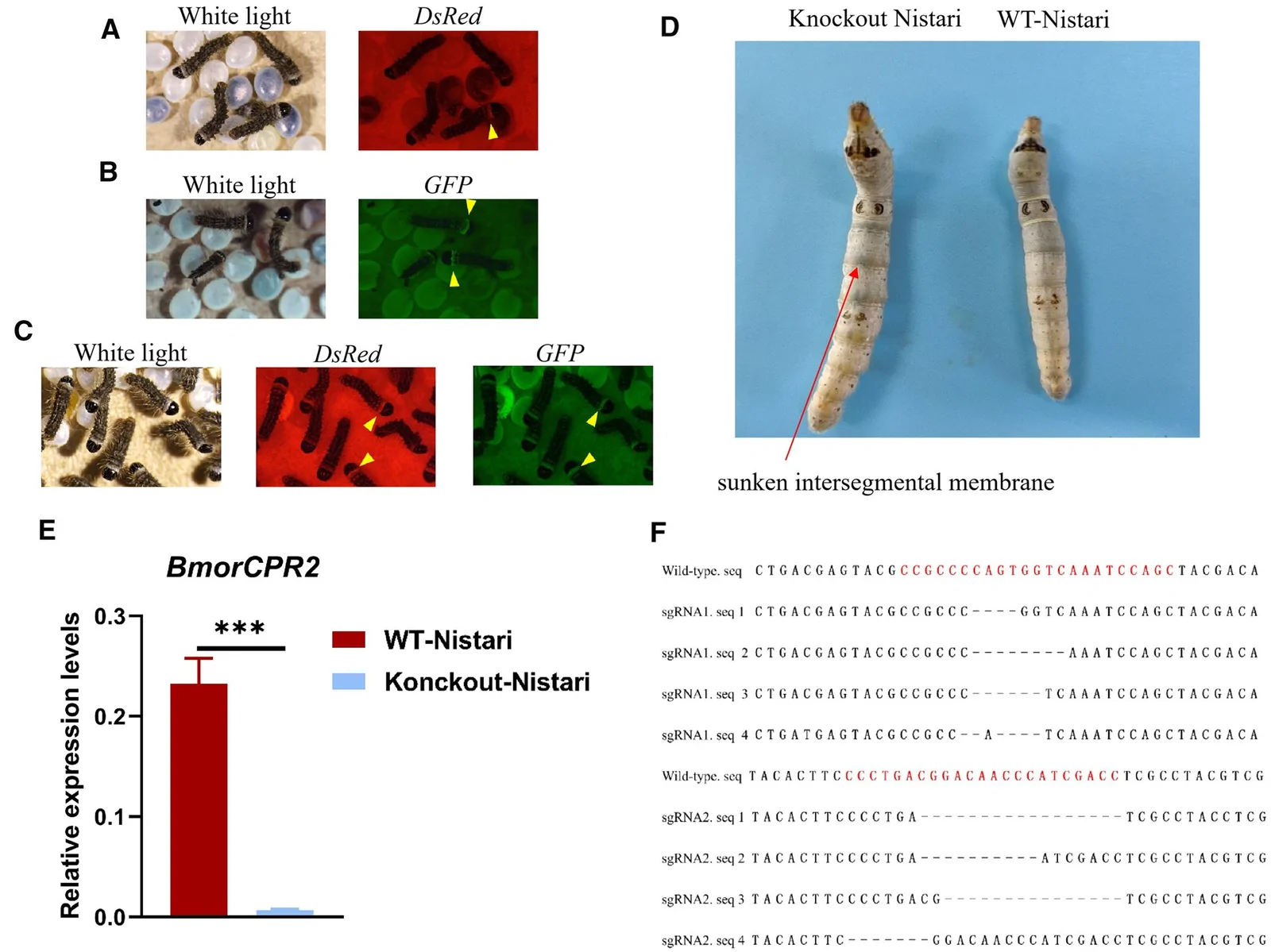
Screening of fluorescent individuals and G_2_-generation wild-type and knockout Nistari. A) Positive G_1_ individual emitting *DsRed*. B) Nistari-Cas9 transgenic individual emitting *GFP*. C) G_2_ dual-fluorescent individual emitting both *DsRed* and *GFP*. Positive individuals are indicated by yellow arrows. D) Phenotype comparison of G_2_-generation wild-type and knockout Nistari. The intersegmental membrane of the knockout individual is sunken. The silkworm stage is on the third day of the fifth instar. E) Relative expression levels of *BmorCPR2* between wild-type and knockout Nistari. Data are presented as mean ± SE from three biological replicates. The expression of *BmorCPR2* in the knockout Nistari is significantly lower than in the wild type. F) CRISPR/Cas9 induces mutation in *BmorCPR2* in G_2_ individuals. Wild-type Nistari is shown, with red sequence representing sgRNA target sites and dashed lines indicating the knockout region.

After microinjection, G_0_ individuals were self-crossed to produce the G_1_ generation. G_1_ larvae were examined under a fluorescent stereomicroscope, and *DsRed* individuals were selected (Fig. 4A). These moths were crossed with a Cas9 transgenic silkworm (Fig. 4B) to generate the G_2_ generation. G_2_ larvae expressing both *DsRed* and *GFP* were selected for knockout validation (Fig. 4C).

To verify *BmorCPR2* function in the silkworm cuticle, two sgRNA constructs were designed targeting exons 2 and 4 of *BmorCPR2* to minimize off-target effects. The G_2_ generation exhibited sunken intersegmental membranes (Fig. 4D), and *BmorCPR2* expression in the knockout Nistari was significantly lower than in the wild-type (Fig. 4E, Supplementary Fig. S3, and Supplementary Table S2). Genomic DNA from five G_2_ individuals with sunken intersegmental membranes was extracted, and sequencing revealed various deletions at the sgRNA target sites (Fig. 4F and sgRNA Knockout sequencing.fasta), indicating the success of the knockout experiment. These results confirmed that *BmorCPR2* is responsible for the sunken intersegmental membrane in *st^^sk^*.

## Discussion

Insect cuticle serves as a critical barrier against pathogens and environmental stress, and plays a key role in body shape and development (Delon and Payre 2004, Moussian et al. 2005). The insect cuticle, composed of chitin and proteins, is vital for growth and adaptability (Liu 2022). In non-silkworm insects, silencing or deleting cuticular protein (CP) genes has led to defective cuticle formation, indicating the importance of CPs in developmental processes (Jasrapuria et al. 2012, Liang et al. 2014, Zhao et al. 2019). This study found that mutation of the cuticular protein gene *BmorCPR2* led to depression of the intersegmental membranes in *Bombyx mori*. This fully demonstrates that the *BmorCPR2* gene is indispensable for the formation of the silkworm cuticle.

Epidermal proteins not only contribute to maintaining the physical properties of the exoskeleton but also participate in regulating important physiological processes such as insect growth and development, environmental adaptation, and immune defense (Stamm et al. 2021). Silencing cuticular protein genes *CPR27* and *CPR18* of *Tribolium castaneum* resulted in structural abnormalities in the adult cuticle (Arakane et al. 2012). The silencing of the *TcCPR27* gene led to disordered protocuticle structure and abnormal channel fibers, resulting in shortened and shrunken elytra; affected adults died prematurely because of dehydration a week after emergence (Noh et al. 2014). Silencing genes encoding 15 CPR family cuticular protein members in *Nilaparvata lugens* (Stal, Hemiptera: Delphacidae) resulted in insect death (Pan et al. 2018). These findings demonstrate that CPR family proteins play an indispensable role in insect cuticle formation as well as growth and development. All members of the CPR family contain the Rebers & Riddiford (R&R) motif. Based on analyses of the R&R conserved motifs, the CPR family is divided into three subfamilies: RR-1 (associated with soft insect cuticles), RR-2 (associated with hard insect cuticles), and RR-3 (associated with sclerotized cuticles) (Andersen 1998, Pan et al. 2018). *BmorCPR2*, identified in this study as controlling *st^^sk^* mutant traits, also belongs to the RR-1 cuticular protein gene of the CPR family. However, the economic traits of the *st^^sk^* mutant did not undergo significant changes.

**Fig. 5.**
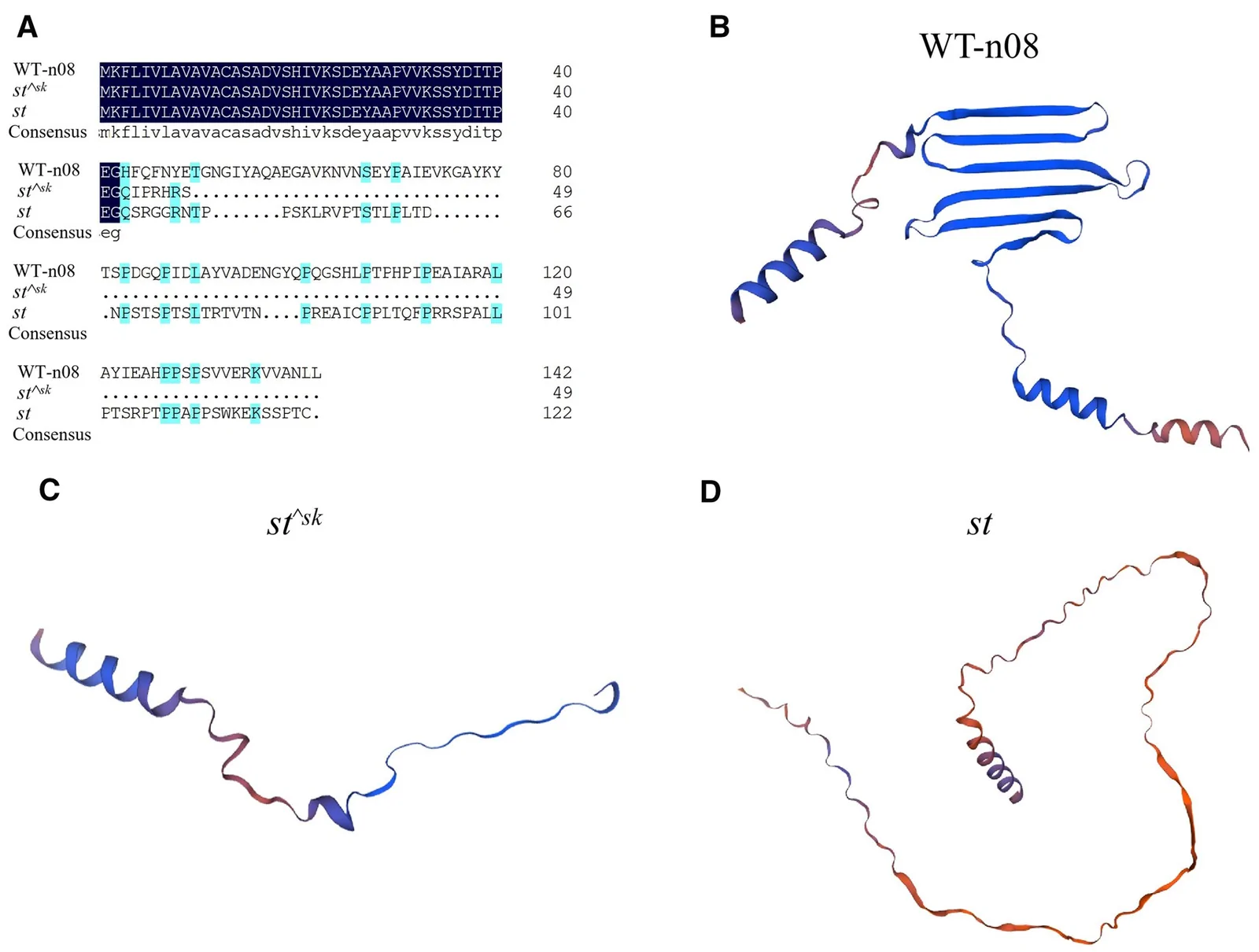
BmorCPR2 protein sequences and the predicted structure diagrams of *st* and *st^^sk^* mutants. A) *BmorCPR2* protein sequences of *st* and *st^^sk^*. B–D) The predicted structure diagrams of *st* and *st^^sk^*.

In a previous study, *BmorCPR2* has been identified as the gene responsible for the body shape mutant *st* (Qiao et al. 2014). A 58 bp deletion and a 5 bp single-nucleotide substitution in exon 2 of *BmorCPR2* in mutant *st* lead to a frameshift mutation and a premature termination codon, resulting in mutant larvae with rigid, tense, and hardened bodies, raised intersegmental folds, and severe adaptive deficiencies (Qiao et al. 2014). This study employed positional cloning and CRISPR/Cas9 gene editing techniques to confirm that the gene controlling the *st^^sk^* mutant trait is also *BmorCPR2*. In the *st^^sk^* mutant, a 73-bp deletion was observed in exon 2 of this gene, along with partial deletions in intron 2, which differ from the mutation sites in the st mutant. *BmorCPR2* encodes a protein of 142 amino acid residues. In both *st^^sk^* and st mutants, different mutation sites lead to frameshift mutations and premature termination codons, with 49 and 122 amino acid residues remaining, respectively (Fig. 5A). According to the website’s (SWISS-MODEL) prediction, by analyzing the structural diagrams of WT-n08, *st^^sk^* and *st* proteins, it can be observed that there are significant differences in the protein structures of the *st^^sk^* and st mutants (Fig. 5B–D). It is speculated that this might be the reason for the emergence of different phenotypes. The marked phenotypic divergence between these mutants (*st^^sk^* and *st*) demonstrates that distinct mutation sites within the same gene can lead to different developmental outcomes in silkworms. These findings lay the foundation for subsequent investigations into the mechanism by which the *BmorCPR2* gene regulates cuticle formation, growth, and development in *B. mori*, thereby providing critical molecular targets for developing novel control strategies against Lepidopteran pests.

## Supplementary Material

ieag089_Supplementary_Data

## Data Availability

Original data from the current study are available in Mendeley Data, doi: 10.17632/r3mzrc5zmd.1. The datasets generated or analyzed during the current study are available in the GenBank repository, accession number: OQ547777.
