# Supplementary material for: Partial deletion in the cuticular protein gene BmorCPR2 results in a body shape mutant in silkworm, Bombyx mori L. (Lepidoptera: Bombycidae)
Source: J Insect Sci. 2026 Aug 21;26(4):ieag089. doi: 10.1093/jisesa/ieag089 (PMC13493190; doi:10.1093/jisesa/ieag089)
Supplement: Supplementary file 1 [file ieag089_supplementary_data.zip › Figure S1 .pdf]

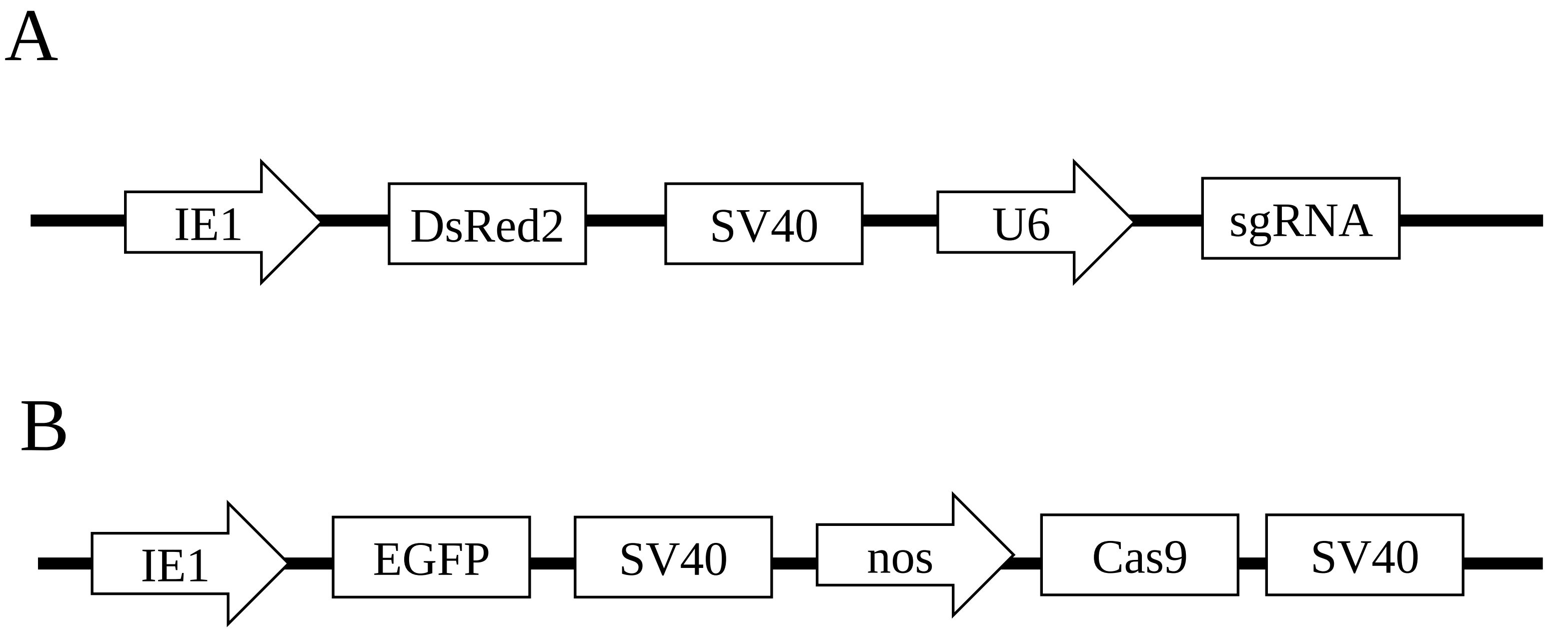
A
IE1
DsRed2
SV40
U6
sgRNA
B
IE1
EGFP
SV40
nos
Cas9
SV40
