# Supplementary material for: Partial deletion in the cuticular protein gene BmorCPR2 results in a body shape mutant in silkworm, Bombyx mori L. (Lepidoptera: Bombycidae)
Source: J Insect Sci. 2026 Aug 21;26(4):ieag089. doi: 10.1093/jisesa/ieag089 (PMC13493190; doi:10.1093/jisesa/ieag089)
Supplement: Supplementary file 1 [file ieag089_supplementary_data.zip › Figure S2 .pdf]

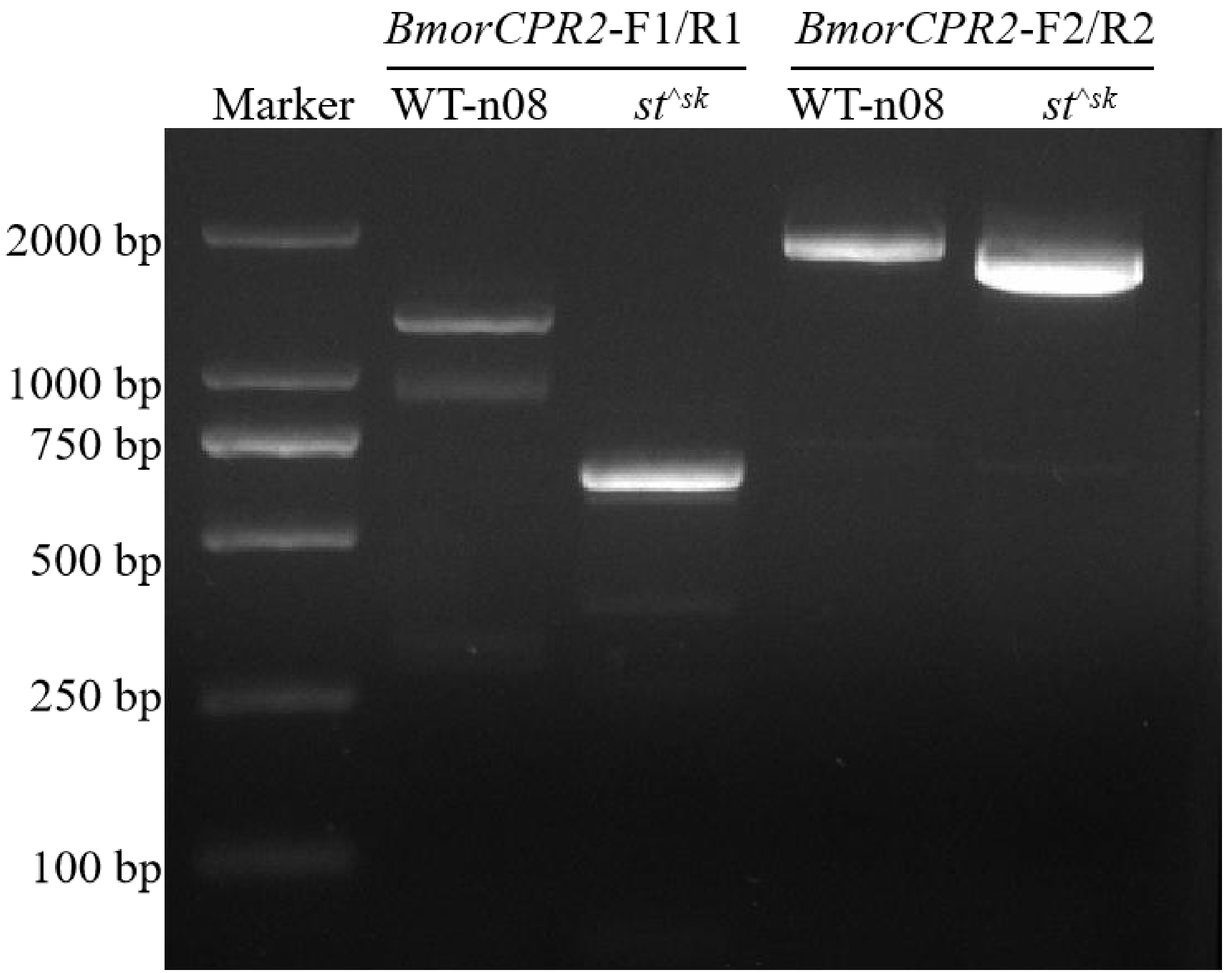
BmorCPR2-F1/R1
BmorCPR2-F2/R2
Marker
WT-n08
st^sk
WT-n08
st^sk
2000 bp
1000 bp
750 bp
500 bp
250 bp
100 bp
