# Supplementary material for: Partial deletion in the cuticular protein gene BmorCPR2 results in a body shape mutant in silkworm, Bombyx mori L. (Lepidoptera: Bombycidae)
Source: J Insect Sci. 2026 Aug 21;26(4):ieag089. doi: 10.1093/jisesa/ieag089 (PMC13493190; doi:10.1093/jisesa/ieag089)
Supplement: Supplementary file 1 [file ieag089_supplementary_data.zip › supplementary information.docx]

**Supporting information**

Table S1. Primers used in gene cloning, functional verification and expression analysis.

Table S2. Raw qPCR Ct values.

Figure S1. The diagrams of the sgRNA and Cas9 building blocks.

Figure S2. PCR products amplified from genomic DNA obtained from *st^^sk^* and WT-n08. Primer sets are shown in (Fig. 3a).

Figure S3. Melt curves of *Actin 3*, *GAPDH* and *BmorCPR2*.

**sgRNA-CRISPR off-target predictions results:**

#Bulge type	crRNA	DNA	Chromosome	Position	Direction	Mismatches	Bulge Size

X	CCCCAGTGGTCAAATCCAGCNRG	CaCCAGTGtTCAAATCCAGCAGG	DF090358.1	196550	-	2	0

sgRNA2-CRISPR off-target predictions results

#Bulge type	crRNA	DNA	Chromosome	Position	Direction	Mismatches	Bulge Size

X	TGACGGACAACCCATCGACCNRG	TGAtGGgCAACCCATgGcCCAAG	DF090367.1	2568527	+	4	0

X	TGACGGACAACCCATCGACCNRG	TGACcGACAACCCcTCaAaCGGG	DF090373.1	1108095	-	4	0

X	TGACGGACAACCCATCGACCNRG	TcACGGACtcaCCATCGACCTAG	DF090417.1	419120	+	4	0

X	TGACGGACAACCCATCGACCNRG	TtACGaAaAACaCATCGACCAAG	DF090320.1	3379663	-	4	0

X	TGACGGACAACCCATCGACCNRG	TGACGttCAACCCATaGACaCAG	DF090331.1	279713	-	4	0

X	TGACGGACAACCCATCGACCNRG	TGgCGGAgAACaCtTCGACCTAG	DF090337.1	4199136	+	4	0

**Bulge type:** X represents no bulge, complete pairing or only mismatches; **crRNA:** The crRNA sequence; **DNA:** The genomic DNA sequence at the predicted potential off-target sites; **Chromosome:** The chromosome number where the potential off-target site is located; **Position:** The start position coordinate of the off-target site on the chromosome; **Direction:** The direction of the DNA strand where the off-target site is located. “+” indicates the normal strand (forward), “-” indicates the antisense strand (reverse); **Mismatches:** The number of base mismatches between crRNA and the DNA sequence. The larger the number, the lower the match to the target sequence, and consequently the lower the risk of off-target cleavage; **Bulge Size:** The size of the bulge (number of bases). A value of 0 represents no bulge.
