# Supplementary material for: Partial deletion in the cuticular protein gene BmorCPR2 results in a body shape mutant in silkworm, Bombyx mori L. (Lepidoptera: Bombycidae)
Source: J Insect Sci. 2026 Aug 21;26(4):ieag089. doi: 10.1093/jisesa/ieag089 (PMC13493190; doi:10.1093/jisesa/ieag089)
Supplement: Supplementary file 1 [file ieag089_supplementary_data.zip › Table S1.docx]

Table S1 Primers used in gene cloning, functional verification and expression analysis

| Genes | Prime name | Primer sequences (5’→3’) |
| --- | --- | --- |
| S0819 | PCR | F: GGCATAATCGCATCGCTCG  R: TCACGAAATGCCAATCATAACTG |
| S0816 | PCR | F: GAAATCCGTTTGAAGAATCCACA  R: CATCCGTTGAATGAGTATCGTTTG |
| S8-4-10 | PCR | F: TGTACTAGGAGACTCCGCT  R: TGATACAGAGGCTATACTGT |
| S8-3-4 | PCR | F: CTCTGCAATATGGTCATCAGT  R: TCATAGAAGCAACAGCCATCT |
| S8-3-9 | PCR | F: GAGTGAGGCTTACGACACGT  R: GATACGAAACGCACTGATCT |
| S8-2-3 | PCR | F: TCGTTGCCGTTCTCGAGTT  R: CAAGGGATGCCTTGTTCCAAT |
| S8-2 | PCR | F: CTGTAAAGATCACGCATTGTCG  R: TAGAAGAGCTCGACGAAGGT |
| *BmorCPR2* | Cloning PCR | F1: CTCCCACATCGTCAAGTCTG  R1: CTAGTAAGAGCAGTGCTTCG  F2: GGGTTAGAGAAGACACAGTC  R2: CAGAGGCTGCTGTAAAGAAG |
| *BmorCPR2*-sgRNA1 | PCR | F: AATATCGTGCTCTACAAGTGAGGAGGGCCTGCATGTTGT GTTTTAGAGCTAGAAATAGC  R: AAGGACCACTACCGAACCACACTTGTAGAGCACGATATT |
| *BmorCPR2*-sgRNA2 | PCR | F: GTGGTTCGGTAGTGGTCCTTGTTTTAGAGCTAGAAATAGC  R: TATAGATATCAAGCTGCTAGAAAAAAAAGCACCGACTCGG |
| *BmorCPR2*- knockout detection | PCR | F1: CTCCCACATCGTCAAGTCTG  R1: CGAAGCACTGCTCTTACTAG  F2: GGGTTAGAGAAGACACAGTC  R2: CTTCTTTACAGCAGCCTCTG |
| *BmorCPR2* | qRT-PCR | F: GTGGTCAAATCCAGCTACGAC  R: GTCTCGTAGTTGAACTGGAAG |
| *A3* | qRT-PCR | F: GGATGTCCACGTCGCACTT  R: GCGCGGCTACTCGTTCACT |
