# Supplementary material for: Partial deletion in the cuticular protein gene BmorCPR2 results in a body shape mutant in silkworm, Bombyx mori L. (Lepidoptera: Bombycidae)
Source: J Insect Sci. 2026 Aug 21;26(4):ieag089. doi: 10.1093/jisesa/ieag089 (PMC13493190; doi:10.1093/jisesa/ieag089)
Supplement: Supplementary file 1 [file ieag089_supplementary_data.zip › Table S2 .docx]

Table S2 Raw qPCR Ct values

|  | *GAPDH* |  | *BmorCPR2* |  |  |  |  |
| --- | --- | --- | --- | --- | --- | --- | --- |
| WT-n08-1 | 18.79 |  | 21.77 |  |  |  |  |
|  | 18.64 |  | 21.78 |  |  |  |  |
|  | 18.8 | 18.74 | 21.79 | 21.78 | -3.03 | 0.121 |  |
| WT-n08-2 | 18.64 |  | 21.76 |  |  |  |  |
|  | 18.81 |  | 21.85 |  |  |  |  |
|  | 18.65 | 18.7 | 21.71 | 21.77 | -3.07 | 0.118 |  |
| WT-n08-3 | 18.57 |  | 21.86 |  |  |  |  |
|  | 18.55 |  | 21.92 |  |  |  |  |
|  | 18.6 | 18.57 | 21.91 | 21.89 | -3.32 | 0.099 | 0.113523562 |
| *st^^sk^-1* | 18.89 |  | 26.92 |  |  |  |  |
|  | 18.72 |  | 26.89 |  |  |  |  |
|  | 18.74 | 18.78 | 26.92 | 26.90 | -8.12 | 0.003 |  |
| *st^^sk^-2* | 18.59 |  | 27 |  |  |  |  |
|  | 18.71 |  | 27.01 |  |  |  |  |
|  | 18.71 | 18.67 | 27.05 | 27.02 | -8.35 | 0.003 |  |
| *st^^sk^-3* | 18.42 |  | 26.57 |  |  |  |  |
|  | 18.46 |  | 26.58 |  |  |  |  |
|  | 18.5 | 18.46 | 26.61 | 26.58 | -8.12 | 0.003 | 0.00340805 |

|  | *Actin 3* |  | *BmorCPR2* |  |  |  |  |
| --- | --- | --- | --- | --- | --- | --- | --- |
| WT-n08-1 | 19.8 |  | 21.77 |  |  |  |  |
|  | 19.85 |  | 21.78 |  |  |  |  |
|  | 19.78 | 19.81 | 21.79 | 21.78 | -1.97 | 0.255 |  |
| WT-n08-2 | 19.67 |  | 21.76 |  |  |  |  |
|  | 19.66 |  | 21.85 |  |  |  |  |
|  | 19.76 | 19.69 | 21.71 | 21.77 | -2.07 | 0.237 |  |
| WT-n08-3 | 19.57 |  | 21.86 |  |  |  |  |
|  | 19.62 |  | 21.92 |  |  |  |  |
|  | 19.63 | 19.60 | 21.91 | 21.89 | -2.29 | 0.204 | 0.232263351 |
| *st^^sk^-1* | 19.91 |  | 26.92 |  |  |  |  |
|  | 19.94 |  | 26.89 |  |  |  |  |
|  | 19.99 | 19.94 | 26.92 | 26.90 | -6.96 | 0.008 |  |
| *st^^sk^-2* | 19.63 |  | 27 |  |  |  |  |
|  | 19.62 |  | 27.01 |  |  |  |  |
|  | 19.59 | 19.61 | 27.05 | 27.02 | -7.40 | 0.005 |  |
| *st^^sk^-3* | 19.38 |  | 26.57 |  |  |  |  |
|  | 19.39 |  | 26.58 |  |  |  |  |
|  | 19.41 | 19.39 | 26.61 | 26.58 | -7.19 | 0.006 | 0.006915897 |
